# The Role of Heme Oxygenase-1 in Remote Ischemic and Anesthetic Organ Conditioning

**DOI:** 10.3390/antiox8090403

**Published:** 2019-09-16

**Authors:** Inge Bauer, Annika Raupach

**Affiliations:** Department of Anesthesiology, University Hospital Duesseldorf, 40225 Duesseldorf, Germany; inge.bauer@med.uni-duesseldorf.de

**Keywords:** heme oxygenase-1, remote ischemic conditioning, anesthetic conditioning, organ protection

## Abstract

The cytoprotective effects of the heme oxygenase (HO) pathway are widely acknowledged. These effects are mainly mediated by degradation of free, pro-oxidant heme and the generation of carbon monoxide (CO) and biliverdin. The underlying mechanisms of protection include anti-oxidant, anti-apoptotic, anti-inflammatory and vasodilatory properties. Upregulation of the inducible isoform HO-1 under stress conditions plays a crucial role in preventing or reducing cell damage. Therefore, modulation of the HO-1 system might provide an efficient strategy for organ protection. Pharmacological agents investigated in the context of organ conditioning include clinically used anesthetics and sedatives. A review from Hoetzel and Schmidt from 2010 nicely summarized the effects of anesthetics on HO-1 expression and their role in disease models. They concluded that HO-1 upregulation by anesthetics might prevent or at least reduce organ injury due to harmful stimuli. Due to its clinical safety, anesthetic conditioning might represent an attractive pharmacological tool for HO-1 modulation in patients. Remote ischemic conditioning (RIC), first described in 1993, represents a similar secure option to induce organ protection, especially in its non-invasive form. The efficacy of RIC has been intensively studied herein, including on patients. Studies on the role of RIC in influencing HO-1 expression to induce organ protection are emerging. In the first part of this review, recently published pre-clinical and clinical studies investigating the effects of anesthetics on HO-1 expression patterns, the underlying signaling pathways mediating modulation and its causative role in organ protection are summarized. The second part of this review sums up the effects of RIC.

## 1. Introduction

**Figure 1:**
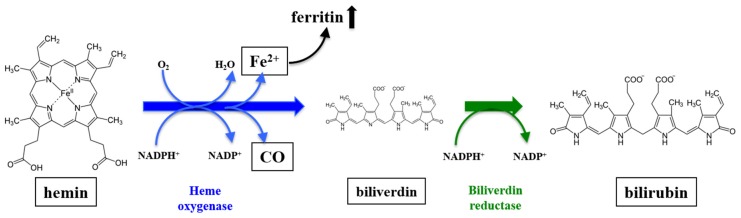
Heme oxygenase pathway. CO: carbon monoxide.

Heme oxygenase (HO) catalyzes the first and rate-limiting step in the degradation of free, pro-oxidant heme [1] (Figure 1). While the isoform HO-2 is almost ubiquitously expressed, the basal, functional expression of the isoform HO-1 (heat shock protein 32) under physiological conditions is mainly restricted to cells involved in erythrocyte degradation; i.e., reticuloendothelial cells of the spleen, bone marrow and liver [2,3,4]. However, HO-1 is highly inducible under numerous environmental stress conditions [4,5,6,7,8,9,10]. Induction of HO-1 under conditions associated with, e.g., inflammation, ischemia/reperfusion (I/R), toxic events or microcirculatory disturbances does not only serve as a biomarker of stress but can be considered as a key cellular adaptive response to such deleterious stimuli. This is supported by the fact, that blockade of the HO-pathway under conditions such as those associated with hemorrhage and resuscitation (H/R), might aggravate organ injury [11]. The protective properties of the HO-pathway are well described and are mainly based on the anti-oxidative, anti-inflammatory, anti-apoptotic, anti-proliferative, anti-thrombotic and vasodilatory effects of the metabolic products—carbon monoxide (CO) and bilirubin, for a long time solely considered toxic waste products [12,13,14,15]—and the induction of ferritin synthesis [16]. The important biological role of endogenously produced metabolic products is strengthened by the fact that protection can be mimicked by exogenous application [14].

Evidence suggests a dual role of HO-1, protective and detrimental [12,15]. Differential effects of heme metabolites are visible on a subcellular level [17]. Furthermore, excessive overexpression might lead to additional cell damage, probably due to a transient accumulation of catalytically active free iron or toxic (local) CO or bilirubin levels that might limit or overwhelm the protective effects [12,18,19]. The amount of metabolic products and the cellular environment seem to determine beneficial or detrimental effects [12,13]. Therefore, the safety and efficacy of various strategies to induce HO-1, like HO-1 gene delivery or the application of (toxic) chemicals, remain a major concern in humans and are mainly limited to pre-clinical studies.

To make the transition into the clinic, further strategies are needed which are effective and ensure a very high level of safety to the patient, ideally, without exerting a general stress response. Recent reviews discuss therapeutic strategies (including the pharmacological application of products of the HO-pathway and of natural dietary antioxidant compounds) to target HO-1 in various disease states [14,20]. A targeted induction of HO-1 by a non-lethal stimulus applied pre- or shortly post-onset of a potentially harmful event might thereby provide an effective strategy to induce tolerance to the tissue at risk, a phenomenon, called “conditioning.” Since the first description of the phenomenon in 1986 [21], several time points of application of the conditioning stimulus have been described, pre- (applicable in predictable events), per- or post- (unpredictable events) onset of the respective stressful stimulus, and there is an early and late window of protection [22].

In the perioperative setting or treatment of patients on the intensive care units (ICU), anesthetics are commonly used for the induction and maintenance of general anesthesia, sedation of patients or pain therapy. Their safety profile is well defined. Clinical trials are published showing that anesthetic conditioning with, e.g., propofol [23] or sevoflurane [24] has protective effects in detrimental events, whereas other trials found neutral effects or recommended further investigations for the respective cohort of patients [25,26]. Indeed, the research is ongoing with eight currently registered clinical trials (www.clinicaltrials.gov (21 August 2019)). Anesthesia-relevant drugs have been shown to modulate the HO-1 system. This topic has been nicely summarized in a review by Hoetzel and Schmidt in 2010 [9]. In the first part of our review, we focus on recently published studies in this field investigating the effects of anesthetics on HO-1 expression patterns, the underlying signaling pathways mediating modulation, and its causative role in organ protection.

With regard to the safety of patients, remote ischemic conditioning (RIC), especially in the non-invasive form, i.e., via a tourniquet/blood pressure cuff applied to a limb, has been intensively investigated in pre-clinical and clinical studies for organ protection in the last few years. Since its first description in 1993 [27], a protective effect by RIC was not only shown for the heart, but also for brain, liver, skin, lung and kidney [28]. Protection against I/R injuries is, meanwhile, widely acknowledged. Recent evidence suggests that RIC is also protective against inflammatory stimuli, such as those associated with sepsis [29]. Currently, the clinical RECO-Sepsis trial started investigating the effect of RIC on septic shock [30]. Numerous clinical trials implied promising results for protection of multiple organs by RIC, but there are also studies with neutral results [28,31,32]. The interest in a translation of RIC into the clinical setting remains high. This is reflected by the fact that currently 90 ongoing clinical trials are registered at www.clinicaltrials.gov (21 August 2019), investigating the potentially protective effect of RIC in diverse diseases. The mechanisms of RIC-induced protection are incompletely understood [28]. Recently, the role of HO-1 in RIC emerged.

In the second part, we focus on the potential role of HO-1 in RIC-induced organ protection.

## 2. The Role of Anesthetic Agents on HO-1 Modulation

Anesthetics are widely used for the maintenance of general anesthesia as well as for sedation of patients in ICU and for pain therapy. Studies suggest pleiotropic effects, including anti-oxidative, anti-apoptotic and immunomodulatory properties leading to cellular protection. Conditioning strategies to induce tolerance against potentially harmful events (e.g., I/R-injury, inflammation) have been evaluated in numerous organs like the heart, brain, kidneys and lungs. Beneficial effects have been described for inhalational anesthetics (volatile anesthetics and the noble gas xenon) in various disease models in vitro and in vivo [33,34,35,36,37,38,39,40,41,42,43,44,45,46]. Cytoprotective effects have also been described for intravenous anesthetics; e.g., morphine [47], propofol [48], ketamine [49] or dexmedetomidine [50,51,52,53]. For both inhalational and intravenous anesthetics, a modulatory role for HO-1 has been suggested [9].

Like for RIC, “classical” preconditioning protocols for inhalational anesthetics exist, comprising one or more cycle(s) of short application periods interspersed with washout or memory phases. However, other conditioning protocols exist, like per or postconditioning, all revealing an early or (extended) late window of protection [41,54].

**Figure 2:**
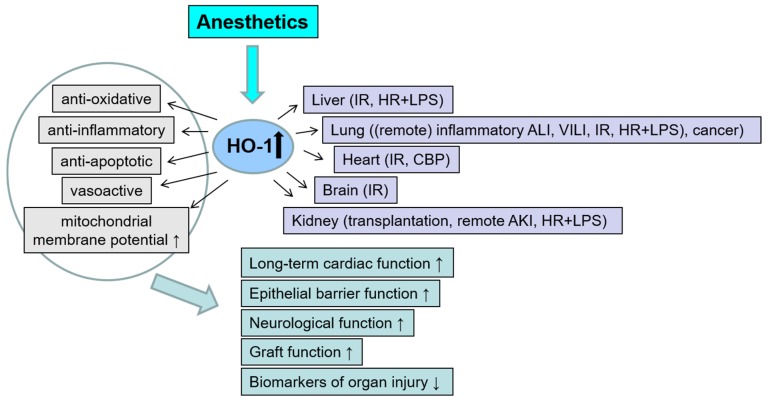
Protective effects of anesthetics. Organs and stress models (purple), mechanisms (grey) and effects on organ injury and function. HO: heme oxygenase; IR: ischemia/reperfusion; HR: hemorrhagic shock; LPS: lipopolysaccharide; ALI: acute lung injury; VILI: ventilator-induced lung injury; CBP: cardiopulmonary bypass; AKI: acute kidney injury.

A detailed description of the conditioning protocols used and further information on the herein-described studies are listed in the supplemental Table S1. Figure 2 summarizes the investigated organs and stress models, the mechanisms of protection and the effects on organ injury and function.

Interestingly, elevated intraoperative carboxyhemoglobin (COHb) levels in patients have been associated with the application of volatile halogenated ether anesthetics. CO is produced during a reaction of desiccated carbon dioxide absorbents within anesthesia machine apparatuses [55,56,57]. Thus, in addition to effects on HO-1 expression resulting in increased production of protective heme metabolites, the release of biologically relevant concentrations of CO might contribute to the cytoprotective mechanisms of these substances [58].

### 2.1. Isoflurane

Isoflurane, a halogenated ether (1-Chloro-2,2,2-trifluoroethyl difluoromethyl ether) exerts general anesthetic and muscle relaxant activities. Isoflurane is used in adult patients; the minimum alveolar concentration (MAC) value is 1.2%. The drug is minimally metabolized (<0.2%) with trifluoroacetic acid as the main metabolite. Main mechanisms of action involve binding to the gamma-aminobutyric acid (GABA)_A_ receptor and the glycine receptor [59,60].

#### 2.1.1. Isoflurane: Preconditioning

The role of isoflurane-induced HO-1 expression in liver I/R has been broadly investigated by Schmidt et al. [61,62]. They nicely show a newly occurring and high expression of HO-1 in hepatocytes and demonstrate the functional role of HO-1 induction for the maintenance of liver integrity and liver macro- and microcirculation in hepatic I/R. It is important to note that HO-1 induction was not accompanied by a general stress/heat shock response in these studies. In a mouse model of ventilator-induced lung injury (VILI), the beneficial effect of isoflurane was, however, associated with a downregulation of HO-1, suggesting a role of HO-1 as a stress marker, but not as mediator of anti-inflammatory and lung-protective effects [63].

#### 2.1.2. Isoflurane: Postconditioning

While previous studies [61,62,64,65] focused on preconditioning, in 2013 Dong et al. published a study using a post-conditioning protocol [66]. Isoflurane, at a clinically relevant concentration (1.4 vol%), was applied for 2 h starting from 6 h after sepsis induction with cecal ligation and puncture (CLP) in male Sprague–Dawley rats. CLP-induced severe sepsis led to acute lung injury (ALI) 24 h after CLP. Isoflurane reduced histopathological changes, capillary protein leakage and pulmonary vascular permeability compared with the control group. However, the extent of lung edema was not affected. Application of isoflurane enhanced CLP-induced HO-1 protein expression. Parallel to reduction of lung injury, isoflurane also suppressed inducible nitric oxide (NO) synthase (iNOS) protein induction, indicating an anti-inflammatory reaction. This might explain, at least in part, the protective effects of isoflurane. Application of Zinc protoporphyrin IX (ZnPP-IX), an inhibitor of HO-activity, reduced the protective effect, indicating a functional role of HO-1 induction in the lung during severe sepsis. Furthermore, ZnPP-IX further increased iNOS expression levels, providing evidence for a mechanistic link between the HO-1- and the iNOS-pathway.

The zymosan-induced generalized inflammation (ZIGI) model closely mimics human multiple organ dysfunction syndrome (MODS) [67], with structural and functional changes in the liver, intestine, lungs, pancreas and kidneys [68]. In an experimental mouse model of zymosan-induced ALI, postconditioning effects of a clinically used isoflurane concentration were tested [69]. 1.4 vol% isoflurane were applied for 1 h, starting 1 h and 6 h after zymosan treatment in a sealed chamber. Isoflurane increased survival. At 24 h after zymosan treatment, lung injury was reduced by isoflurane, as reflected in lower pulmonary edema and histological changes. Inflammatory markers and signs of apoptosis were also reduced in the lungs. These protective changes were also visible in bronchoalveolar lavage (BAL) samples. Here, total cell number and polymorphonuclear leukocytes (PMNs) were reduced. This was also paralleled by a reduced inflammatory response at the protein level. In addition, isoflurane further increased zymosan-induced HO-1 mRNA, protein and activity in lung tissue, suggesting the participation of HO-1 in protection [69]. These results are in line with a study by Konrad et al. showing that HO-1 induction with cobalt protoporphyrin IX (CoPP-IX) diminished PMN migration into the alveolar space after lipopolysaccharide (LPS) inhalation, which was associated with an anti-inflammatory response [70].

Besides anesthetic doses, subanesthetic concentrations of isoflurane have also been investigated in the context of cytoprotection in zymosan-induced lung injuries. Wang et al. provides a study with in vitro and in vivo experiments on the protective effects of isoflurane postconditioning in lungs [71]. In the in vitro series, isolated type II alveolar epithelial (AE II) cells were treated with 0.5 mg/mL zymosan. After 30 min, cells were incubated for 30 min in a metabolic chamber in the presence of 0.7 vol% (subanesthetic) isoflurane. Twenty-four hours after the beginning of the zymosan-challenge, isoflurane diminished lactate dehydrogenase (LDH) activity, tumor necrosis factor alpha (TNF-α) and interleukin-1 beta (IL-1β) production, and apoptosis in wild type AE II cells. HO-1 protein and activity, and phosphorylated signal transducer and activator of transcription (p-STAT3) protein were further increased by isoflurane. Experiments with small interfering RNA (siRNA)-induced HO-1 or STAT3-silencing or adenovirus-induced overexpression of HO-1 or STAT3 suggest a functional role in protection and a positive feedback loop comprised of STAT3 and HO-1 [71].

These results were confirmed in vivo in the same experimental model of zymosan-induced ALI as used by Li et al. [69]. Wild type (wt) and HO-1-deficient (HO-1^-/-^) male BALB/c mice were used to clarify the functional role of HO-1 [71]. In wt mice, 0.7 vol% isoflurane reduced mortality, lung edema, histological changes and pulmonary cell apoptosis in the lungs. In BAL, the total number of cells and proinflammatory cytokine levels were reduced. Zymosan-induced HO-1 mRNA, protein and activity, along with STAT3 protein, were further increased in the lungs. In HO-1-deficient mice, protective effects of isoflurane were abolished [71]. In summary, HO-1/STAT3 signaling mediates the protection of subanesthetic isoflurane against zymosan-initiated lung injury.

Thus, also postconditioning with isoflurane with either subanesthetic or anesthetic concentrations seems to exert cytoprotective effects. The use of subanesthetic concentrations of isoflurane especially provides an attractive therapeutic approach extending its use beyond surgical procedures. More studies are warranted to confirm these observations and elucidate the exact functional role of HO-1 under these conditions.

#### 2.1.3. Isoflurane: Perioperative Application

In neuronal injury or neurodegenerative disease, both neuroprotective and neurotoxic effects of isoflurane are discussed [45,49]. Recently, Fang et al. described an in vivo rat model of lumbar spinal cord ischemia (SCI) by cross-clamping of the thoracic aorta at the level of T5 [72]. Ischemia was performed for 25 min, followed by cord reperfusion for up to 48 h. Isoflurane 1.5 vol% was applied via inhalation perioperatively. SCI-induced motor dysfunction significantly deteriorated with isoflurane. This effect was associated with a neurodegenerative process and apoptosis in the spinal cord. Myeloperoxidase (MPO) levels remained unchanged. SCI-induced HO-1 protein expression was further induced by isoflurane. This increase was interpreted as an enhanced inflammatory response [72].

These data provide further evidence for a potential neurotoxic effect of isoflurane. HO-1 induction might be considered as part of the inflammatory response to SCI. Whether, e.g., tissue-specific effects or the mode of application perioperatively versus pre- or postconditioning determine beneficial or rather detrimental effects of isoflurane, remains to be determined.

### 2.2. Sevoflurane

Sevoflurane (1,1,1,3,3,3-Hexafluoro-2-(fluoromethoxy) propane) is used in children and adults for induction (0.5–8% (high amounts in children)) and maintenance (0.5–3%) of general anesthesia. In middle-aged adults, the MAC value is 2%. Sevoflurane exerts profound hypnotic effects, while muscle relaxant and analgesic effects are rather weak. The metabolization rate is about 3–5% with inorganic fluoride, carbon dioxide and hexafluoroisopropanol as main metabolites. Like isoflurane, it mainly acts via binding to GABA_A_ receptors and glycine receptors [59,60,73].

#### 2.2.1. Sevoflurane: Preconditioning

In a recent study by Zhang et al., the effect of sevoflurane preconditioning on myocardial infarction was investigated. Myocardial infarction was induced in male Sprague–Dawley rats by a 30 min left anterior descending artery (LAD) occlusion with 2 h of reperfusion [74]. Sevoflurane preconditioning was performed using 2.5 vol% for 30 min before ischemia. Sevoflurane improved cardiac function and reduced infarct size, and LDH and creatine kinase (CK) releases as markers of injury. Serum malondialdehyde (MDA) levels decreased while superoxide dismutase (SOD)-activity increased, thus counterbalancing the oxidative stress response. The systemic inflammatory response was also attenuated. In addition, signs of apoptosis were reduced. In this study, microRNA (miR)-374 expression was investigated. MiRs are small non-coding RNAs which negatively regulate gene expression at the posttranscriptional level and play essential roles in regulating cell differentiation, proliferation and apoptosis [75]. While miR-374 expression increased, the expression of one of its target genes, the transcription factor SP1, decreased on the mRNA and protein levels, suggesting a regulatory role of miR-374. Signaling pathways (phosphatidyl inositol 3-kinase (PI3K)/protein kinase B and (Akt)/glycogen synthase kinase 3 beta (GSK3ß)) were activated; p53, iNOS, c-fos mRNA and protein expression decreased. Sevoflurane enhanced I/R-induced HO-1 mRNA and protein expression. In the light with additional results in primary cardiomyocytes transiently transfected with SP1-siRNA, mir374- mimic or inhibitor, the authors propose that miR-374 might represent an interesting therapeutic target regulating the transcription factor SP1 through activating the PI3K/Akt pathway [74].

The exact functional role and the mechanism of upregulation of HO-1 under these conditions remain to be determined.

Lee et al. used a “classical” preconditioning protocol with washout phases to study the protective effects of sevoflurane on brain I/R injuries [76]. Sprague–Dawley rats were subjected to transient global cerebral ischemia with bilateral common carotid artery occlusion. After ischemia, two periods of inhalation of 2 vol% sevoflurane for 10 min, followed by a washout period of 10 min were applied. At days 1 and 7 after reperfusion, markers of brain injury and expression levels of HO-1 and translocation of the nuclear factor—erythroid-2-related factor 2 (Nrf2) to the nucleus were assessed [76]. Nrf2 is considered a master regulator of antioxidant genes. HO-1 has been described as a downstream target of the transcription factor Nrf2. The mechanisms of HO-1 expression involve enhanced Nrf2-translocation to the nucleus. Nrf2 regulates antioxidant response element (ARE)-driven HO-1 expression [77,78]. Sevoflurane improved neurological function, and reduced signs of necrosis and apoptosis 1 d and 7 d after injury. In the early phase, sevoflurane post-conditioning increased I/R-induced nuclear Nrf2 and cytosolic HO-1 protein expression. A blockade of protein kinase C (PKC) signaling by chelerythrine reversed the effects of sevoflurane on Nrf2 and HO-1 expression and on neuroprotection. These results suggest a regulatory role of the transcription factor Nrf2 in the induction of HO-1 and support data on a major role of the Nrf2/HO-1 signaling pathway in neuroprotection after cerebral I/R injury [76]. A direct link to the HO-pathway and to the functional role of HO-1 in conferring protection was not provided.

#### 2.2.2. Sevoflurane: Postconditioning

Next to sevoflurane preconditioning, numerous studies investigated effects of sevoflurane postconditioning on HO-1. Ye et al. applied sevoflurane postconditioning (2.5 vol% sevoflurane) for 60 min with onset of reperfusion using a more severe model of focal cerebral ischemia with 60 min of bilateral common carotid artery occlusion in male Sprague–Dawley rats [79]. Twenty-four hours after reperfusion, sevoflurane decreased infarct size, neuronal injury and signs of apoptosis. I/R-induced HO-1 mRNA and protein expression were further increased by sevoflurane. The effect of sevoflurane alone was not tested. Messenger RNA and protein of the upstream target hypoxia induced factor-1 alpha (HIF-1α) were upregulated in parallel, indicating a regulatory role of HIF-1α. The causal relationship between HIF-1α/HO-1 and PI3K/Akt was investigated by a PI3K/Akt blockade with wortmannin. Wortmannin partly blocked the protective effects and reduced HO-1/HIF-1α -induction [79].

As mentioned above, there is evidence, that the degree of HO-1 expression determines whether an intervention is rather beneficial, or even detrimental. Using a model of hemorrhagic shock with a 45 min hemorrhage (35–40 mmHg) and different time periods of resuscitation (H/R) in male Sprague–Dawley rats, such a dual neuroprotective-neurotoxic role was elegantly investigated [80]. Sevoflurane postconditioning was performed for 5 min after onset of resuscitation using a clinically common (2 vol%) and a supraclinical (4 vol%) concentration. While postconditioning with 2 vol% improved learning ability, reduced apoptosis and ROS-production and increased mitochondrial membrane potential levels after H/R in hippocampal tissue; 4 vol% sevoflurane had no protective effect or even worsened H/R-effects. Administration of 2 vol% sevoflurane increased H/R-induced HO-1 protein expression and activity in hippocampal tissue. This effect was even more pronounced with 4 vol% sevoflurane. Pretreatment with hemin further induced HO-1 expression and activity and reversed the protective effects of 2 vol% sevoflurane. Tin protoporphyrin IX (SnPP-IX) reduced HO-1 expression and activity and partially reversed the neurodegeneration of 4 vol% sevoflurane [80]. These results support the hypothesis that exceeding a certain threshold of HO-activity aggravates injury or reverses effects of a protective intervention. Whether the potentially negative effects of high (local) amounts of heme metabolites or tissue-specific properties are responsible, needs to be elucidated.

Besides conditions of oxidative stress (I/R-injury, VILI), possible protective effects of sevoflurane have also been investigated in inflammatory conditions. Zhao et al. investigated the effect of sevoflurane postconditioning and the regulatory role of HO-1 in LPS-induced acute lung injury in male Sprague–Dawley rats [81]. Inhalation of sevoflurane (2.4 vol%), started 2 h after LPS (5 mg/kg intravenous (i.v.)) and continuing for 4 h until the end of the experiment, reduced lung injury. This was shown in diminished edema formations and pathomorphological lung injury scores. Furthermore, inflammatory markers were reduced. Sevoflurane shifted the pro-/antioxidant balance to an anti-oxidant state as reflected in decreased MDA levels and increased SOD activity in lung tissue. While sevoflurane alone did not affect HO-1 expression levels, additive effects on LPS-induced HO-1 mRNA and protein expression were observed. HO-1 induction was mediated by PI3K/Akt. Pretreatment with LY294002, a PI3K inhibitor, prior to LPS, reduced HO-1 expression and partially reversed the protective sevoflurane effects. The authors concluded that sevoflurane alleviates ALI through the induction of HO-1 via the PI3K/Akt pathway [81]. The study underpins data from a previous study on sevoflurane postconditioning: Zheng et al. showed that sevoflurane activates the PI3K/Akt pathway to protect isolated rat hearts against I/R-induced apoptosis [82].

Patients suffering from diabetes are more susceptible to myocardial I/R injury. There is evidence that endogenous adaptive and conditioning effects can be blocked by various confounders like age and comorbidities [83,84]. Furthermore, diabetes and (acute) hyperglycemia seem to also interfere with these protective mechanisms ([83,85,86,87,88]. Interestingly, acute hyperglycemia itself, when induced prior to or during ischemia, has been shown to reduce myocardial I/R-injury [89].

The effect of high glucose levels on sevoflurane postconditioning in the context of HO-1 was investigated in vitro and in vivo by Gao et al. [90]. Healthy male C57BL/6 mice and mice with streptozotocin (STZ)-induced diabetes were subjected to 45 min LAD occlusion with 2 h of reperfusion. Postconditioning was induced with the inhalation of 2 vol% sevoflurane during the first 15 min of the coronary reperfusion period. In normal mice, sevoflurane reduced infarct size, the release of CK-MB and LDH, oxidative stress and apoptosis. Furthermore, nuclear Nrf2 expression was further increased. Diabetes aggravated myocardial I/R-injury and abolished sevoflurane-induced protective effects. HO-1 protein expression increased in healthy mice, while diabetes abolished sevoflurane-induced HO-1 induction. Blockade of the HO-pathway with ZnPP-IX abolished protective effects of sevoflurane and partly inhibited Nrf2-translocation and HO-1 induction [90]. Similar results were obtained in vitro in H9c2 cardiomyocytes subjected to 3 h of hypoxia (1% O_2_) with 6 h of reoxygenation in normal (5.5 mM) or high (25 mM) glucose containing cell culture media, using the same postconditioning protocol. Under normal glucose, sevoflurane increased cell viability and attenuated release of LDH into the culture medium. Antioxidant capacity was restored. Hyperglycemia aggravated hypoxia/reoxygenation-induced cell damage and abolished sevoflurane effects. HO-1, as well as nuclear Nrf2 expression, were increased under normoglycemic, but not under hyperglycemic culture conditions. The inhibition of HO-activity reversed the protective effects of sevoflurane under normoglycemia [90]. These results suggest a functional role of HO-1 in sevoflurane-induced protection against myocardial I/R-injury and a participation of Nrf2 as an important regulator of HO-1. This underpins the important role of Nrf2 as the master regulator of the antioxidant defense response which seems to be limited under the conditions of hyperglycemia.

Most studies focus on either pre- or postconditioning effects. Thus, it is interesting to directly compare the efficacy of both forms. Shiraishi et al. subjected male Wistar rats to partial liver ischemia for 1 h with 3 h of reperfusion [91]. Sevoflurane preconditioning was performed by applying 2.5 vol% sevoflurane for 30 min starting 35 min before ischemia. The postconditioning protocol encompassed 2.5 vol% sevoflurane for 30 min starting 5 min before reperfusion. Sevoflurane reduced liver injury, as reflected in reduced aspartate aminotransferase (AST), alanine aminotransferase (ALT) and LDH-activities, and histological changes. Pre- and postconditioning were equally protective. Both treatments affected HO-1 expression pattern in the liver as shown in increased expression in Kupffer cells (KC). ZnPP-IX partially inhibited the protective effects, indicating a functional role of HO-1 in KC in liver I/R [91].

#### 2.2.3. Sevoflurane: Continuous Application

While numerous studies using pre- or postconditioning protocols demonstrate protective effects, the impact of continuous application during potentially harmful stimuli is not clear [92,93]. The ventilation of male C57BL/6N mice with tidal volumes of 16 mL/kg for 4 h induced lung injury in comparison to controls (8 mL/kg) [94]. Sevoflurane 1 vol% was continuously applied during ventilation, which reduced VILI. In the lungs, histological score decreased and the mRNA of nuclear factor ‘kappa-light-chain-enhancer’ of activated B-cells (NF-κB), an important regulator of the inflammatory response, was downregulated. Likely as a result of impaired NF-κB-signaling, in BAL, levels of proinflammatory cytokines TNF-α, IL-1ß and IL-6 decreased, while the anti-inflammatory IL-10 increased. Inhalation of sevoflurane led to a further increase of high tidal volume-induced HO-1 mRNA expression in lung tissue [94]. These results are in contrast to the study by Faller et al. using isoflurane as a protective agent in a similar model of VILI, where protection was associated with a downregulation of HO-1. Since heat shock protein (HSP) 70 was concomitantly downregulated, this was interpreted as reduced stress response by isoflurane [63]. It needs to be confirmed, that the increased HO-1 mRNA expression by mechanical stimulation, observed by Xiong et al. [94], leads to an increase in HO-1 protein and activity and participates in reduced local cytokine formation.

Independent of the application protocol, sevoflurane is organ protective and increases HO-1 levels. This HO-1 induction should be well adapted to avoid high, potentially detrimental levels of HO-1 activity. Nrf2 and PI3K/AKT signaling seem to be main mediators of sevoflurane induced HO-1 expression. Under diabetic conditions, protection of sevoflurane is reduced probably caused by inhibited increase of HO-1 gene expression and activity.

### 2.3. Xenon

Xenon is a noble gas with hypnotic and analgesic properties and very favorable characteristics, including the preservation of hemodynamic stability, rapid awakening and a favorable safety profile [95,96,97,98]. Thus, xenon is an emerging anesthetic and might be advantageous over other anesthetics, especially in (cardiovascular) patients at risk or during specific interventions [99]. The main mechanism of action consists of non-competitive inhibition of the excitatory glutamate signaling, mainly via *N*-methyl-d-aspartate (NMDA) receptor [96].

So far, there is only one study available investigating xenon in the context of HO-1 regulation [100]. It is an elegant experimental in vivo study using a cold I/R injury model of renal transplantation, complemented with an in vitro study using a model of hypothermia/hypoxia with reoxygenation, simulating the transplantation process. In an orthotopic iso- and allograft transplantation model, rats were treated in an anesthetic chamber with 70% xenon. Either donor rats were treated 24 h prior to kidney retrieval for 2 h, investigating the late window of protection or recipient rats received xenon immediately after engraftment (postconditioning). Treatment of the donor or recipient with xenon decreased the number of terminal deoxynucleotidyl transferase dUTP nick end labeling positive (TUNEL+) cells and improved the function of kidney grafts. The inflammatory response was reduced, as reflected in diminished NF-κB activation in cortical tubular epithelial cells of transplanted grafts. Major histocompatibility complex class II (MHCII) expression on tubular cells was, likewise, reduced. Morphological and functional deterioration associated with acute rejection were attenuated. Xenon enhanced HO-1 protein expression in the renal cortex of donor rats. In parallel, heat shock protein-70 (HSP70) was upregulated. Similar results of xenon pre- and postconditioning on viability and apoptosis were observed in vitro in human proximal tubular HK-2 cells subjected to hypothermia/hypoxia with reoxygenation. HO-1 siRNA almost completely abolished HO-1 protein induction and reduced cell viability, indicating a causal relationship. HSP70-siRNA also reversed the protective effects [100].

These results suggest that HO-1 induction by xenon is not a targeted effect under the studied conditions but rather part of a general stress/heat shock response. Thus, protection seems to be mediated by the concerted action of both HSPs. The underlying mechanisms by which xenon, as a presumed inert gas, triggers a general heat shock response, is unknown.

### 2.4. Morphine/Opioids

Morphine exerts strong analgesic effects with the central nervous system as its central site of action. The drug can be applied via different routes, preferably i.v. and intrathecal (i.t.). Morphine acts mainly as a reversible agonist on µ-opioid receptors, while binding activity to Ƙ receptors is rather weak.

#### 2.4.1. Morphine: Preconditioning

The role of HO-1 in morphine-induced effects under physiological conditions in vitro and in vivo was studied by Patel et al. in 2003 [101]. For the in vitro study, they used a mouse macrophage cell line (J774) as a model system of immune cells. Cells were pretreated with different clinically relevant concentrations of morphine (10^−14^–10^−6^ M) for 16 h. Morphine reduced cell migration and induced an apoptotic response. Pretreatment with morphine induced HO-1 protein expression and HO-activity, with the strongest effect at middle concentrations. An HO-1 inducer, hemin, mimicked the morphine effect and further enhanced morphine-induced effects. Inhibition of HO-activity with ZnPP-IX attenuated these effects, indicating a functional detrimental role of HO-1 in this experimental model. These results could be confirmed in vivo in mice pretreated with morphine (20 mg subcutaneous (s.c.), daily for 10 d). Migration of macrophages to the peritoneal cavity was reduced by morphine despite enhanced chemoattraction and the number of apoptotic cells strongly increased versus control. HO-1 expression levels were not assessed. However, experiments with hemin and ZnPP-IX confirmed a participation of HO-1. These results suggest a rather harmful impact of HO-1 induction by morphine. As a potential mechanism, the authors proposed that the morphine-mediated HO-1 induction leads to a reduction in ROS, which are required for bacterial killing [101].

In contrast to the above described study, cell protective effects of morphine have been described in the context of HO-1 induction [102]. Induction of HO-1 has previously been shown to protect against liver I/R injury in normal [103] and cirrhotic [104] livers. Male Sprague–Dawley rats, healthy or with carbon tetrachloride-induced liver cirrhosis, received different concentrations of morphine either intrathecally (0.1, 1 or 10 µg/kg) or intravenously (1, 10 or 100 µg/kg) 10 min prior to 1 h partial warm liver ischemia with 6 h of reperfusion. In normal and cirrhotic liver, morphine preconditioning at the highest concentrations protected against I/R-injury as reflected in lower histological injury score, ALT and AST-activities, signs of necrosis and apoptosis. Both routes of morphine application induced HO-1 expression in cirrhotic liver after I/R; expression patterns in normal liver were not assessed. Furthermore, in normal liver, this protective morphine effect required activation of peripheral µ-opioid receptors and of various pro-survival signaling pathways (PI3K/Akt). In cirrhotic liver, activation of Akt, PKC alpha/betaII and inhibition of iNOS expression seems to be involved. Effect of blockade of HO-activity was not investigated.

Morphine exerts similar protective effects on IR injury in normal and cirrhotic rat liver, however with distinct activation of downstream pathways [102]. The exact functional role of HO-1 in morphine-induced protection against liver IR-injury needs further investigation.

#### 2.4.2. Morphine/Opioid: Postconditioning

The effect of morphine in postincisional pain and inflammation was investigated by Godai et al. using a mouse model of hindpaw plantar incision [105]. Two different concentrations of morphine (3 μg/20 μL or 10 μg/20 μL) were injected locally to the site of incision either early (at 1 h after the skin was sutured and at postoperative days (POD) 1 and 2) or late (at PODs 5–7). Pain behavior was assessed and it revealed that mechanical hyperalgesia was reduced on PODs 7, 12 and 14, but only with early morphine application, whereas the latency to a thermal stimulus and paw edema remained unchanged. While IL-1ß mRNA was markedly decreased on POD 7, TNF-α mRNA levels were not affected. Gene expression of the endogenous opioid, proenkephalin, was increased on POD 7. Observed changes were associated with a phenotype shift of local macrophages through a decrease in pro-inflammatory F4/80^+^iNOS^+^ M1 macrophages and an increase in wound healing F4/80^+^CD206^+^ M2 macrophages (POD 2 and 7). Morphine seems to act on the recruitment and activation of macrophages during the early phase after injury, since prior depletion of local macrophages reversed the analgesic effects of morphine on mechanical hyperalgesia and the macrophage switch [105]. HO-1 has been described as the promotor of differentiation of macrophages to the M2 phenotype [106]. Paw incision upregulated local mRNA expression levels of HO-1, with no additional effects of morphine. Unfortunately, protein expression was not investigated. Inhibition of HO-activity with tin protoporphyrin-IX (SnPP-IX) led to a significant decrease of the mechanical threshold and reversed the shift in macrophage phenotypes induced by morphine, indicating a causal relationship. Since HO-1 mRNA expression was not altered by morphine, the authors concluded that μ-opioid receptor signaling may be downstream of HO-1, or that HO-1 induction itself is independent of μ-opioid receptor signaling in macrophages [105].

Beside morphine, the regulatory role of selective receptor agonists on HO-1 has been studied. Tong et al. investigated acute and long term effects of a selective κ-opioid agonist on myocardial I/R-injury [107]. Male Sprague–Dawley rats were subjected to myocardial ischemia for 30 min with 24 h (acute) and with up to 4 weeks (long term) of reperfusion. A single dose of U50, 488H (2 mg/kg), a selective κ-opioid agonist, was applied 5 min before reperfusion. Administration of U50, 488H exerted both acute (reduction in infarct size) and long-term beneficial effects with improved cardiac function and remodeling. In addition, oxidative stress was diminished. U50, 488H upregulated myocardial HO-1 mRNA (up to 48 h) and protein (up to 6 d) after I/R. Pharmacological inhibition of HO-activity with ZnPP-IX reversed acute and long-term protection. Investigation of Nrf2 translocation and the pharmacological inhibition of PI3K provide evidence of a PI3K–Akt–Nrf2 pathway that mediates U50488H-induced beneficial effects and HO-1 transcription and expression. In neonatal rat cardiomyocytes in vitro, I/R was simulated. U50, 488H was applied during simulated reperfusion and could reduce apoptosis. Consistent with the in vivo results with pharmacological inhibition, HO-1 shRNA abolished the reduction in apoptosis. The upstream signaling was not investigated in vitro. The authors concluded, that the PI3K–Akt–Nrf2 pathway mediates the induction of HO-1 expression, which is critical for the prevention of cardiomyocyte death and improved functional recovery by U50, 488H [107].

For morphine postconditioning in an inflammatory pain model, the role of HO-1 is not completely clear and needs further investigation. In contrast, postconditioning with the selective κ-opioid U50, 488H, reveals a functional role for HO-1 in mediating acute and long-term cardiac protection.

### 2.5. Propofol

Propofol, 2,6–diisopropylphenol, is a short acting substance used for the induction and maintenance of general anesthesia (adults and children older than 1 month); sedation for diagnostic and surgical interventions (alone or in combination with local or regional anesthesia); and sedation of intubated, mechanically ventilated adult (>16 y) patients on ICU. Propofol mediates its effects mainly via a positive modulation of GABA_A_ receptors [59,108,109].

The potential role of HO-1 is studied in various organs and cells (lungs, kidneys liver, heart and brain) and is focused on several signaling cascades, like Nrf2 and mitogen-activated protein kinase (MAPK) under several propofol conditioning protocols.

#### 2.5.1. Propofol: Preconditioning

Yao et al. subjected rats to orthotopic liver autotransplantation (OLAT) [110]. Rats were intraperitoneally injected with propofol at two different concentrations, termed low (L: 50 mg/kg) or high (H: 100 mg/kg) as pretreatment for three consecutive days before OLAT. Remote injury to the lungs (ALI) was investigated 8 h after OLAT. Propofol preserved lung morphology with a more pronounced effect at the higher dosage. Pulmonary edema was reduced. Furthermore, oxidative stress was dose-dependently reduced, as shown in lower levels of hydrogen peroxide (H_2_O_2_) and MDA, which were accompanied by higher SOD activity. The high concentration of propofol showed moderate additive effects on OLAT-induced HO-1 protein expression in lung tissue. The regulatory role of Nrf2 was studied and confirmed. The levels of cytosolic Kelch-like ECH-associated protein 1 (KEAP1) protein, a negative regulator of Nrf2, were decreased with parallel induction of nuclear Nrf2; NAD(P)H quinone dehydrogenase (NQO1), like HO-1, a downstream target of Nrf2, was increased with both concentrations of propofol [110].

Interestingly, low dose propofol was still protective against OLAT-induced ALI, but without inducing HO-1 over OLAT levels. Furthermore, low dose propofol exerted similar effects on the regulation of KEAP1, Nrf2 and NQO1 as high dose. Thus, the differential regulation of HO-1 by L- and H-dose propofol in response to OLAT needs further evaluation.

In the same experimental model of OLAT and propofol pretreatment the role of the Nrf2/HO-1 pathway was also studied in protection against acute kidney injury (AKI) [111]. Results obtained in the lungs by Yao et al. [110] could be confirmed for the kidneys. Propofol protected the kidney, as shown in reduced kidney pathology, serum injury markers blood urea nitrogen (BUN) and creatinine (Crea), along with a reduction in oxidative stress in kidney tissue. High propofol further upregulated OLAT-induced HO-1 protein expression. As described for the lungs, at the high concentration, the Nrf2/HO-1/NQO1 pathway could provide a potential underlying mechanism of protection. Furthermore, consistent with results in the lungs, low propofol also exerted protective effects against AKI but without increasing HO-1 versus OLAT [111].

Thus, further mechanisms seem to be involved in propofol preconditioning, especially with lower concentrations, in OLAT-induced remote injuries to the lungs and kidneys.

Xu et al. investigated effects of propofol preconditioning in hyperglycemia-induced myocardial injury in vitro and in vivo [112]. Primary cardiomyocytes, isolated from neonatal rats were cultured in high glucose (25.5 mM, HG) containing medium for 48 h. Propofol 50 µM was applied 30 min before high glucose exposure. Propofol attenuated cardiomyocyte hypertrophy, increased viability and reduced apoptosis. Furthermore, signs of oxidative stress were reduced. These effects could be attributed to the drug, since the solvent intralipid had no effect. Interestingly, HG reduced HO activity, while increasing HO-1 protein. Propofol restored HO-activity and further increased HO-1 protein expression. Induction of HO-1 with cobalt protoporphyrin showed similar effects as propofol. ZnPP-IX, an HO-inhibitor, reversed propofol effects. The functional role of HO-1 could be confirmed by HO-1 siRNA experiments in H9c2 cells [112].

Next, they tested whether the cardioprotective effects of propofol on hyperglycemia-induced cardiomyocyte damage were also visible in vivo in type I diabetes rats. After 8 weeks of diabetes propofol was infused i.v. for 45 min at 900 μg/kg/min. Cardiac dysfunction was attenuated by propofol infusion. Propofol decreased plasma and myocardial free 15-F_2t_-isoprostane levels as markers of oxidative stress. Furthermore, pro-apoptotic factors were reduced, while anti-apoptotic B-cell lymphoma 2 (Bcl2) was increased. Propofol did not significantly alter HO-activity and HO-1 expression in control hearts. Similar to the results obtained in vitro in HG treated cardiomyocytes, in diabetic heart: HO-activity was decreased despite parallel upregulation of HO-1 protein levels compared to healthy controls. Propofol partly restored activity without further increasing protein levels [112]. Posttranscriptional regulation of HO-1 under HG conditions, resulting in uncoupling between HO-1-expression and HO-activity might represent the underlying mechanism explaining these observations [113,114].

The additional investigation of STAT3 activation suggests that propofol restores hyperglycemia/diabetes-induced reduction in STAT3 phosphorylation. Thus, the HO-1/STAT3 signaling pathway plays a major role in mediating cellular protection and improving cardiac function under conditions of hyperglycemia [112].

Shinjo et al. applied propofol pre- and perconditioning in vitro and in vivo to study myocardial IR-injury [115]. H9c2 cardiac myoblast cells were cultured under oxidative stress conditions, induced by H_2_O_2_ (250 µM) for 24 h. Cells were pretreated with 100 µM propofol for 30 min and for a further 24 h after the addition of H_2_O_2_. LDH-release decreased, reflecting an improved cell viability. The number of TUNEL+ cells decreased, providing evidence for reduced apoptotic effects. While propofol alone had no effect on HO-1 expression, it showed a synergistic effect on H_2_O_2_-induced upregulation of HO-1 mRNA and protein. Furthermore, the role of Nrf2 as an important regulator of HO-1 expression was studied. Nuclear localization of Nrf2 was increased by propofol under oxidative stress. This effect was inhibited by Nrf2-siRNA. Nrf2-siRNA also attenuated induction of NQO1, while HO-1 induction was only slightly and not significantly reduced. In addition, Nrf2-siRNA blocked protective propofol effects. In contrast, HO-1-siRNA and NQO1-siRNA did not block propofol-induced cytoprotection [115].

These results suggest that Nrf2 activation is critical for propofol-induced cytoprotection, but the role of the downstream targets HO-1 and NQO1 is not completely clear.

In the same study, rats were subjected to myocardial ischemia by LAD occlusion for 30 min with 15 or 90 min of reperfusion. Propofol infusion at 22 mg/kg/h was started 10 min after LAD occlusion and continued until the end of reperfusion. No significant HO-1 mRNA induction could be detected at 15 or 90 min after the onset of reperfusion in penumbra or infarction region. Nrf2 level in the penumbra region but not in the infarct region, was elevated early (15 min) after reperfusion [115].

These in vitro and in vivo results provide evidence for an important role of Nrf2 activation under conditions of oxidative stress in cardiomyocytes. The fact that HO-1-siRNA did not block beneficial propofol effects in vitro and no significant HO-1 induction could be observed in vivo should lead us to a rather careful interpretation of the exact functional role of the downstream antioxidant enzyme HO-1. Furthermore, it is important to note, that the study population consisted of only three in vitro, and three or four in vivo, per group.

Besides the Nrf2/HO-1 axis, activation of MAPK seems to play a role in propofol-induced cytoprotection. Human endothelial cells (HUVEC) were cultured under conditions of mild oxidative stress, induced by 100 µM H_2_O_2_, a concentration that only moderately induced HO-1 mRNA and protein [116]. Cells were cultured in the absence or presence of different propofol concentrations (10, 25, 50 µM; dissolved in dimethyl sulfoxide (DMSO)) for up to 24 h. Propofol attenuated H_2_O_2_-induced apoptosis. Under conditions of oxidative stress, propofol dose- and time-dependently upregulated HO-1 mRNA, protein and activity. Inhibition of HO-activity with ZnPP-IX partially reversed the anti-apoptotic effects of propofol. To further elucidate the regulation of HO-1 activation, the role of upstream MAPK-pathways was investigated. Propofol differentially activated MAPK: Propofol activated extracellular signal–regulated kinases (ERK), but not c-Jun N-terminal kinases (JNK) and p38 MAPK, and an ERK-blockade with PD98059 reduced HO-1-induction. Apoptosis was not assessed after the ERK-blockade. These results suggest that, under oxidative stress conditions, propofol induces HO-1 expression in HUVECs and this effect is mediated, at least in part, via the ERK pathway [116].

Similar results were obtained in neuronal cells and the role of the ERK pathway was confirmed. Gu et al. used SHSY5Y, a neuroblastoma cell line, as model system for neuronal tissue [117]. Oxidative stress was induced with 1 mM H_2_O_2_ (a 10× higher concentration as used by Liang et al. [116] for 24 h. Cells were pretreated with propofol at different concentrations (25–100 µM) for 8 h before incubation with H_2_O_2_ in fresh medium. Propofol dose-dependently increased viability and decreased intracellular ROS formation. Propofol upregulated HO-1 protein under conditions of oxidative stress. Interestingly, H_2_O_2_ alone did not affect HO-1 expression; the effect of propofol alone was not tested. The regulatory role of MAPK-was investigated. In said study, propofol increased the phosphorylation statuses of ERK, JNK and p38 MAPK. Pharmacological blockade of ERK (but not JNK or p38 MAPK) reversed propofol-mediated effects on protection and HO-1 upregulation. Thus, in line with the results obtained by Liang et al. [116]), a modulation of HO-1 expression by ERK activation was observed. Furthermore, a functional role of HO-1 in mediating protection was shown [117].

#### 2.5.2. Propofol: Postconditioning

Ge et al. provided a comprehensive study, a clinical trial, animal and cell culture experiments investigating the role of the Brahma-related gene 1 (Brg1)/Nrf2/HO-1 axis in propofol postconditioning in hepatic IR-injury [23]. The first part is a study in patients suffering from hepatic hemangiomas scheduled for orthotopic liver transplantation (OLT). General anesthesia was rapidly induced with a combination of midazolam, sufentanil, propofol and cisatracurium, and maintained with sevoflurane, sufentanil. Postconditioning was performed with injection of propofol (2 mg/kg i.v.) within 10 min after onset of reperfusion in OLT. The main results are that in the early post-operative phase propofol reduced markers of liver injury (AST and ALT) in serum and histological examination of the liver revealed less severe pathological scores. Furthermore, oxidative stress was attenuated, as reflected in reduced serum H_2_O_2_ and MDA levels. However, SOD activities in serum remained unchanged. Propofol upregulated HO-1 protein expression in the donor liver. In parallel, translocation of Nrf2, a known regulator of HO-1 was strongly increased. Furthermore, expression of another important Nrf2 downstream target and antioxidant enzyme, NQO1, was upregulated by propofol. Propofol partially prevented OLT-induced downregulation of Brg1. These results suggest protective effects of propofol mediated via the Brg1/Nrf2/HO-1 axis [23].

In the same study, Ge et al. used an in vivo mouse model to deeper investigate the mechanisms of propofol-induced protection against liver I/R injury. Mice were subjected to partial (70%) warm liver ischemia (60 min) with 6 h of reperfusion. For postconditioning, 40 mg/kg i.p. propofol was applied with the onset of reperfusion. Like in patients, propofol exerted hepatoprotective and antioxidant effects as shown with reduced AST, ALT, histological scores and 8-isoprostane levels in the liver. Propofol upregulated HO-1 protein and restored Brg1 expression. These effects can be attributed to propofol, since the lipid solvent had no effects on the studied parameters. In Brg1-transgenic mice I/R-induced Brg1 downregulation was prevented; Nrf2 translocation and HO-1 protein expression were induced. In an in vitro model of hypoxia/reoxygenation in the human hepatocyte cell line LO2, the important role of the Brg1/Nrf2/HO-1 signaling pathway in mediating propofol-induced protection in hepatic IR-injury could be nicely confirmed in experiments using Brg1 and/or Nrf2 RNA interference. These results suggest that propofol postconditioning attenuates hepatic I/R-injury via Brg1 mediated Nrf2/HO-1 transcriptional activation [23].

Neuroprotective effects of propofol and the role of HO-1 were also investigated in vivo. Liang et al. investigated the effects of propofol postconditioning against brain I/R injury [118]. In male Sprague–Dawley rats the middle cerebral artery was occluded (MCAO) for 90 min followed by 24 h reperfusion. Propofol postconditioning was performed with the application of different doses of propofol (10, 20 and 50 mg/kg/h), dissolved in 10% DMSO, at the onset of reperfusion for 30 min. The higher doses, 20 and 50 mg/kg/h improved neurological outcome, reduced brain infarct size and volume, as well as signs of apoptosis. In combination with MCAO, propofol dose-dependently upregulated HO-1 protein (starting from 20 mg/kg/h) and activity (starting from 10 mg/kg/h) in the ischemic penumbra and core. The effects of MCAO alone on HO-1 expression versus sham were not assessed. Inhibition of HO-activity with ZnPP-IX partially reduced HO-activity and partially reversed the neuroprotective effects. Application of ZnPP-IX alone did not aggravate injury. Hemodynamics remained stable in all groups [118]. Interestingly, although the lowest concentration of propofol significantly increased HO-activity, protective effects of propofol could only be shown with the higher concentrations without differences between the groups.

#### 2.5.3. Propofol: Perioperative Setting

Besides classical conditioning studies, protective effects have also investigated in the perioperative setting with propofol as part of the anesthetic regime. Xia et al. investigated and compared the effects of propofol and midazolam in children undergoing cardiopulmonary bypass (CPB) because of congenital heart disease [119]. Patients received anesthesia with intravenous propofol as hypnotic agent (induction: 1 mg/kg; maintenance: 150 µg/kg/min) or midazolam (induction: 0.1 mg/kg; maintenance: 1 µg/kg/min). Tracheal extubation time and the length of the ICU stay were shorter in the propofol versus midazolam group. Furthermore, markers of inflammation and oxidative stress were reduced at the investigated time points, 2 h after aortic cross-unclamping and 24 h postoperatively. Immunohistochemistry revealed a higher degree of HO-1 protein induction over time in myocardial samples from the right ventricular outflow tract of patients receiving propofol versus midazolam. Thus, protective effects of propofol might be mediated by a stronger HO-1 induction [119].

These studies provide evidence for a functional role of HO-1 in propofol-induced organ protection by pre and postconditioning in vivo under conditions associated with IR and in vitro in H_2_O_2_-induced oxidative stress. Nrf2 and ERK-signaling seem to be the main mechanisms in HO-1 regulation. Furthermore, first indications exist that propofol might exert protection via HO-1 induction during its use for general anesthesia in patients and might be superior over, e.g., midazolam.

### 2.6. Ketamine

Ketamine, a chiral phenylcyclohexylamine derivative with hypnotic and strong analgesic effects is used for induction and maintenance of general anesthesia—preferably for short term diagnostic and surgical procedures, supplementation of regional anesthesia, and general anesthesia and analgesia in emergency medicine. Different routes of administration exist, including i.v., intramuscular (i.m.) and intranasal. The racemic mixture consisting of (*S*) and (*R*)-ketamine has been in clinical use since 1970. Since 1997, S-Ketamine has been commonly used because of its higher potency over R-ketamine (4:1). S-Ketamine exerts complex pharmacological actions, including inhibition of biogenic amine uptake, and binding to opioid receptors and the allosteric blockade of NMDA receptors, whereas the latter is mainly responsible for the anesthetic and analgesic properties [59,120,121].

Recent studies on cytoprotective effects of ketamine and the role of the HO-1 pathway are scarce. Tan et al. focus on LPS-induced inflammation in isolated mouse peritoneal and alveolar macrophages [122]. Cells were pretreated for 12 h with non-toxic concentrations of ketamine (10, 100 and 1000 µM) prior to stimulation with 1 µg/mL LPS for 18 h. While 10 µM had no effect, 100 and 1000 µM dose-dependently reduced the inflammatory response as reflected in decreased cellular mRNA levels of high mobility group box 1 (HMGB1), iNOS, TNF-α and IL-1ß, and concomitant reduction in the release of these markers into the culture medium. Ketamine pretreatment alone without LPS at 100 and 1000 µM upregulated HO-1 protein. In addition, Nrf2 nuclear translocation was enhanced, while NF-κB-activation was diminished (only assessed in peritoneal macrophages). Nrf2 siRNA abolished HO-1 induction. Both SnPP-IX and HO-1-siRNA partially reversed ketamine-induced reduction of proinflammatory markers. Furthermore, SnPP-IX attenuated the inhibitory effect of ketamine on NF-κB-activation [122].

Activation of the Nrf2/HO-1 pathway and HO-1-mediated suppression of NF-κB activation seem to mediate ketamine effects in this in vitro model of inflammation.

These promising results observed in vitro need to be confirmed and extended and translated into pre-clinical animal models.

### 2.7. Dexmedetomidine

The drug dexmedetomidine ((S)-4-[1-(2,3-dimethylphenyl) ethyl]-3H-imidazole) is used for short-term sedation and analgesia (<24 h) of patients in the ICU. Dexmedetomidine represents a new class of highly selective agonist of α2-adrenergic receptors. In addition to sedative effects, analgesic sparing effects, reduced delirium and agitation, perioperative sympatholysis, cardiovascular stabilizing effects and the preservation of respiratory function have been attributed to dexmedetomidine. Dexmedetomidine is available for i.v. application [123].

Dexmedetomidine-mediated pre- and postconditioning in the context of HO-1 was studied in the lungs, kidneys and liver in pre-clinical models and in patients:

Gao et al. investigated dexmedetomidine-mediated preconditioning on the lung in lung cancer patients undergoing elective pulmonary lobectomy with one-lung ventilation (OLV) [124]. Dexmedetomidine was administered at 1 μg/kg 20 min before induction of general anesthesia. TNF-α and MDA serum levels were lower, while SOD serum levels were higher compared to control. Dexmedetomidine administration was associated with higher HO-1 protein expression in the excised pathological lung tissue versus no treatment [124].

Dexmedetomidine effects were also studied in inflammation. A recent study by Feng et al. investigated the effects of dexmedetomidine preconditioning on LPS (10 mg/kg i.p.)- induced kidney injury in male Sprague–Dawley rats [125]. 30 min before LPS, animals received dexmedetomidine (25 µg/kg i.p.) and 4 h after LPS systemic and local effects on injury and markers of oxidative stress and inflammation were assessed. Dexmedetomidine reduced histological markers of kidney injury (haematoxylin and eosin staining) and improved kidney function (reduced biomarkers in serum (BUN, Crea) and urine (kidney injury molecule-1 (KIM-1) neutrophil gelatinase-associated lipocalin (NGAL)). The systemic inflammatory response was attenuated (IL-1ß, TNFα, IL-6 and IL-18). Oxidative stress in the kidney was reduced by dexmedetomidine: A decrease in ROS and MDA was accompanied by an increase in glutathione levels and SOD and catalase activities. HO-1 mRNA and protein were upregulated in renal tubules. Dexmedetomidine promoted LPS-increased phosphorylation of GSK3ß (resulting in inhibition of activity) as well as Nrf2 translocation to the nucleus. Furthermore, NQO1-mRNA levels increased. A GSK3β inhibitor, SB216763, alone showed similar effects like dexmedetomidine on protection and on GSK3ß/Nrf2/HO-1/NQO1 signaling and did not negatively influence dexmedetomidine-effects on the studied parameters [125].

The proposed mechanism was shown to be receptor-dependent, since both atipamezole, an α2-adrenoceptor inhibitor, and idazoxan (imidazoline I2 receptor antagonist) reversed dexmedetomidine effects on the signaling pathway and on protection.

Jiang et al. investigated dexmedetomidine postconditioning in a two hit model of global I/R and LPS-induced inflammation. Multiple organ dysfunction was induced by hemorrhagic shock (60 min 40–50 mmHg) with 60 min reperfusion followed by LPS (15 mg/kg i.v.) injection 60 min after resuscitation [126]. Immediately after LPS administration, dexmedetomidine was applied at 1 µg/kg, i.v. over 10 min followed by 5.0 µg/kg per hour until the end of the experiment at 6 h after onset of shock. Dexmedetomidine improved survival rate and diminished lung injury and renal and liver dysfunction. Prooxidant (MDA) and proinflammatory mediators (TNF-α, IL-1ß, IL-6, IL-8 and NO) decreased, while the antioxidant (SOD) and anti-inflammatory (IL-10) response was enhanced. Dexmedetomidine increased H/R-LPS-induced HO-1 protein expression in the lungs, liver and kidneys. HO-1 induction and protective effects were partly reversed by pharmacological blockade of α2-adrenergic receptors with yohimbine, indicating an—at least in part—HO-1 and receptor-mediated protection [126]. Pre- and postconditioning with dexmedetomidine is protective and induces HO-1 expression. This protection seems to be mediated by similar signaling pathways involving α2-adrenergic and imidazoline I2 receptors.

## 3. The Role of Remote Ischemic Conditioning on HO-1 Modulation

Murry et al. first described ischemic preconditioning, a phenomenon where a pre-treatment with short, non-lethal cycles of I/R reduced the detrimental effects of a global ischemia in hearts of dogs [21]. In this study, four cycles of 5 min circumflex coronary artery occlusion, separated by 5 min reperfusion were performed, followed by 40 min global ischemia. This procedure reduced infarct size to about 25% versus control. To date, the organ protective effects of ischemic preconditioning are widely demonstrated both experimentally and clinically [127]. In 1993, it was shown that the conditioning stimulus given at a remote tissue or organ was also protective for the target organ, a phenomenon called “remote ischemic conditioning” (RIC) [27]. The first report on remote organ conditioning was related to the kidney, where cycles of I/R at the renal artery reduced infarct size in the heart after I/R injury [128]. Meanwhile it is known that one or more short cycles of ischemia and reperfusion by using a tourniquet at a limb are suitable to induce organ protection. Due to its low risk and simplicity, this non-invasive form of RIC, not involving surgical procedures with direct vessel clamping, has advantages in patients. Recent reviews nicely summarize protective effects of RIC on the heart [129], brain [130] and kidneys [131]. The early investigations on RIC focused on application time points for RIC intervention prior to harmful events like ischemia (Pre-RIC). However, RIC intervention after induction of injury has also shown to be effective (Post-RIC).

**Figure 3:**
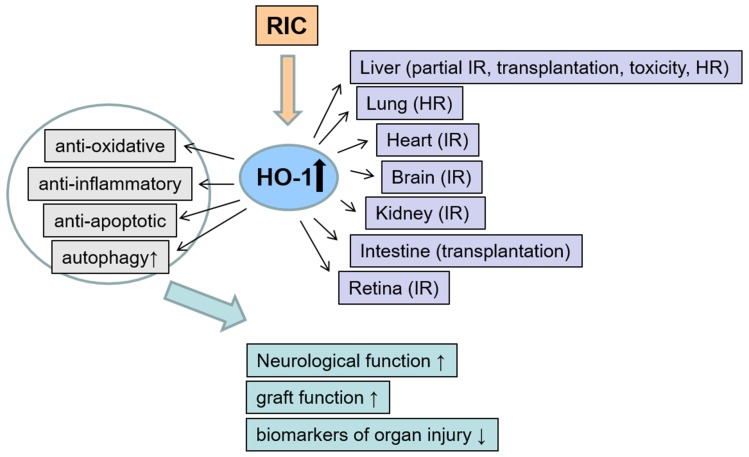
Protective effects of remote ischemic conditioning (RIC). Organs and stress models (purple); mechanisms (grey); and effects on organ injury and function. RIC: remote ischemic conditioning; HO: heme oxygenase; IR: ischemia/reperfusion; HR: hemorrhagic shock with resuscitation.

Since 2014, the number of publications on RIC arises and the first study dealing with Post-RIC was published. The described studies for Pre- and Post-RIC in the context of HO-1 are listed in the supplemental Table S2. Figure 3 summarizes the investigated organs and stress models, the mechanisms of protection by RIC and the effects on organ injury and function.

### 3.1. Remote Ischemic Preconditioning (Pre-RIC)

The investigation of the regulation and function of HO-1 in the context of RIC has only recently attracted interest, starting with a study investigating Pre-RIC in 2006. Since 2014, the number of publications rose and they are categorized under organ-specific subtitles.

#### 3.1.1. Liver

The first study investigating the effect of Pre-RIC on HO-1 was published by Lai et al. in liver I/R injury in rats [132]. They could show that invasive hind limb Pre-RIC protected against liver damage initiated by partial hepatic I/R and increased HO-1 expression. The invasive Pre-RIC protocol of four cycles 10 min femoral artery occlusion followed by 10 min of reperfusion was effective in reducing the I/R-induced increase of the liver injury marker ALT. Analysis of HO-1 expression revealed that Pre-RIC induced HO-1 mRNA and protein levels 4 h after reperfusion in the liver, and that the increase in protein levels persisted throughout 24 h. In contrast, lymphocytes did not show a change in HO-1 levels 4 h after RIC. The activity of HO was induced also by Pre-RIC, whereas the HO-inhibitor, zinc-protoporphyrin (ZnPP-IX), abolished this effect and reversed the RIC-induced hepatoprotection. Thus, the authors concluded that Pre-RIC protects against I/R-induced liver injury by inducing HO-1 expression [132].

Wang et al. underlined the findings of Lai et al. [132] with a similar Pre-RIC protocol to protect the liver in mice [133]. The invasive femoral vascular bundle clamping with four cycles of 4 min I/R prior to warm liver I/R injury reduced liver damage. Similar to the observation of Lai et al. [132], the expression of HO-1 protein increased 2 h after RIC and peaked at 12–24 h, whereas the HO-inhibitor ZnPP-IX abolished these effects. Furthermore, ZnPP-IX did not only abrogate the RIC-induced protection of the liver, but also aggravated I/R injury. In addition, the authors tested the hypothesis that RIC-induced HO-1 expression regulates autophagy to protect the liver against I/R injury. Autophagy seems to play a protective role during liver I/R injury by eliminating damaged or dysfunctional mitochondria to prevent release of toxic mediators like pro-apoptotic signals or reactive oxygen species [134]. Corresponding to the RIC-induced increase of HO-1 expression, autophagy was induced. Again, ZnPP-IX abrogated this effect. The relationship between HO-1 and autophagy was further investigated in an in vitro model simulating I/R by mineral oil. The induction of HO-1 by hemin increased autophagy and phosphorylation of p38 MAPK, but decreased apoptosis. Conversely, HO-1 inhibition with HO-1 siRNA resulted in opposite effects. The results from the in vitro model indicate that HO-1 levels may influence autophagy in a p38 MAPK-dependent pathway for protecting against injury. Thus, RIC induced protection of the liver after I/R seems to be mediated by HO-1/p38 MPAK-dependent autophagy [133].

Also in stress models of hepatotoxicity, like in acute liver failure induced by acetaminophen (APAP) overdose, a similar protocol of invasive hind limb Pre-RIC has therapeutic potential [135]. Zheng et al. showed reduced hepatotoxicity via Pre-RIC with clamping of femoral bundle (four cycles of 5 min ischemia followed by 5 min reperfusion) directly before APAP application in mice by inhibiting the oxidative stress and inflammatory response. Protein levels of HO-1 were elevated by Pre-RIC. Application of an HO-inhibitor, ZnPP-IX, counteracted the positive effects of RIC, whereas the HO-1 inducer cobalt protoporphyrin IX could mimic the reducing effect of RIC on ALT and AST levels as markers of liver damage. Interestingly, the authors directly compared Pre- and Post-RIC (starting immediately after APAP application) showing similar efficacy of protection [135].

Like Zheng et al. [135], Czigany et al. could also demonstrate a protective effect on the liver for Pre-RIC and Post-RIC in a direct comparison, but for Post-RIC to a weaker extent [136]. A different RIC protocol was used with clamping of the infrarenal aorta for four cycles x 5 min I/R directly before or after a stress model of arterialized orthotopic liver transplantation (OLT) in rats. The Pre-RIC in the recipient rats reduced the detrimental effects of I/R injury including tissue damage, apoptosis, graft circulation, inflammation and tissue energetic status of OLT. The HO-1 mRNA expression in the recipient rats was induced 15–20 fold after reperfusion for 3 h, whereby Pre-RIC further increased HO-1 levels. Interestingly, Post-RIC applied after graft reperfusion did not induce a further increase of HO-1. This suggests that Pre- and Post-RIC are mediated by different pathways [136].

A successful protection of the liver against I/R injury by delayed Pre-RIC was described by Kageyama et al. [137]. They performed two cycles of 4 min occlusion of the superior mesenteric artery with 11 min reperfusion followed by total hepatic ischemia after 48 h of recovery. Pre-RIC attenuated hepatic damage and pro-inflammatory cytokine production and improved animal survival. In a second approach, the authors analyzed HO-1 mRNA and protein levels over time after RIC without ischemic injury. In the liver, HO-1 mRNA levels were significantly increased 2 h after RIC and returned to control levels after 24 h. Correspondingly, HO-1 protein levels peaked between 6 and 24 h and returned to basal levels after 48 h. These changes of HO-1 expression levels are similar to the findings of Lai et al. and Wang et al. [132,133]. In comparison to liver, HO-1 mRNA levels in the ileum were very low, but increased also after 2 h of RIC and returned to basal levels after 12 h. However, HO-1 protein levels were not affected by RIC in the ileum. To localize the HO-1 protein in the liver, an immunohistological staining was performed confirming a strong expression in Kupffer cells, which was intensified by RIC [137].

Leung et al. showed hepatoprotective effects using a non-invasive limb Pre-RIC protocol in a model of hemorrhagic shock and resuscitation (S/R) [138]. To characterize the underlying mechanisms, the authors investigated the influence of the HO-1 transcriptional activator Nrf2 on RIC-induced hepatocellular protection by comparing wt and nuclear factor erythroid 2-related factor (Nrf2) knock out (KO) mice. RIC was performed before hemorrhage by occlusion of the left hind limb by tightening a tourniquet for four cycles of 5 min I/R. This treatment reduced hepatocellular injury and enhanced the S/R induced increase of HO-1 mRNA and protein levels. In line with the results of Wang et al. [133], an increase of autophagy after S/R in Pre-RIC treated animals over untreated animals was detected. These results indicate that HO-1-induced autophagy seems to be not only involved in protection against liver I/R injury, but also in an S/R setting. Because Nrf2 expression as well as translocation to the nucleus were increased by RIC, they analyzed the impact of ERK1/2, an upstream regulator of Nrf2 phosphorylation, in protection via RIC. Inhibition of ERK1/2 with PD98059 abolished the RIC-induced increase of HO-1 and Nrf2, and the hepatocellular protection. In Nrf2-KO mice, RIC-induced hepatocellular protection and increase of HO-1 protein levels were abrogated. Interestingly, plasma transfer from RIC-treated wt animals to animals undergoing S/R was able to induce hepatocellular protection in wt but not in Nrf2 KO recipient mice. Plasma from RIC-treated Nrf2 KO mice also failed to induce protection in wt mice undergoing S/R. These results suggest that Nrf2 is necessary to generate protective factors as well as to mediate the protection at the remote organ. HO-1 as a direct target of Nrf2, might also have a potential role in mediating protection, although HO-1 expression was not investigated directly in this experimental approach [138].

#### 3.1.2. Lung

A hemorrhage/resuscitation (H/R) rat model combined with a similar Pre-RIC regime as that by Leung et al. [138] was used by Jan et al. [139]. In this study, the authors focus on the lungs as target organ. Non-invasive hind limb Pre-RIC with three cycles of 10 min I/R were efficient to reduce oxidative stress, inflammatory response and lung injury induced by H/R. HO-1-levels and activity were induced by H/R and Pre-RIC alone, and the combination of both treatments showed additive effects. The authors concluded that a therapeutic effect of induced HO-1 is only given by a so called “super-induction.” H/R alone also increased HO-1 levels as adaptive response to stress, whereas a further increase of HO-1 levels by additional RIC treatment resulted in protection. This was underlined by treatment with an HO-inhibitor; SnPP-IX reversed the protective effects of RIC in H/R induced lung injury, despite a further increase of HO-1 expression. Interestingly, the Pre-RIC induced increase of HO-activity was inhibited SnPP-IX. The authors explained the increase of HO-1 levels by SnPP-IX as a secondary, compensatory effect due to the reduction of HO-activity.

#### 3.1.3. Heart

A study from Zhou et al. investigated whether the number of Pre-RIC stimuli has an influence on delayed cardioprotection [140]. One stimulus of four cycles of 5 min I/R at the hind limbs 24 h prior to ischemia reduced cardiac damage and increased HO-1 mRNA and protein levels. Additional daily RIC stimuli at 2 d and 3 d before ischemia intensified the protective effects and the HO-1 upregulation dependent on the number of stimuli. These results indicate that delayed Pre-RIC is cardioprotective in a dose- and probably HO-1-dependent manner [140].

In a following study this group used the most effective delayed Pre-RIC protocol of three daily stimuli in a direct comparison to postconditioning with 3% sevoflurane bubbled buffer for 10 min at the onset reperfusion (SpoC) [141]. Both forms of conditioning reduced infarct size and cardiac troponin I levels in rat hearts in an isolated, perfused I/R injury model (Langendorff system), but combination of both methods showed additive effects of cardioprotection. A combination of sevoflurane postconditioning with PI3K-inhibitor LY294002 and delayed Pre-RIC with ZnPP-IX abolished the cardioprotective effects. Interestingly, activation of Akt, and not ERK1/2, seems to play only a role during SpoC, whereas HO-1 levels seem to be important for both methods. Both forms of conditioning alone induced HO-1 mRNA and protein levels. The rise of HO-1 levels was abolished by both inhibitors, but further increased by combination of both conditioning methods. The same expression pattern was found for Nrf2 levels, which is in line with the results of Leung et al. [138]. The authors suggested that the additive effect by both forms of conditioning is realized by enhanced HO-1 expression, which is mediated at least partly via Nrf2 translocation [141].

#### 3.1.4. Kidney

An induction of HO-1 levels associated with an increase of Nrf2 levels by Pre-RIC like in the heart was also described in kidneys by Hussein et al. [142]. The authors investigated the effect of hind limb Pre-RIC with three interspersed cycles of ischemia (for 5 min) and reperfusion (for 5 min) on I/R-induced kidney injury. Next to a reduction of kidney damage, RIC impaired expression of anti-apoptotic and pro-inflammatory cytokine genes. Along with results obtained by Lai et al. [132] and Wang et al. [133], HO-1 mRNA levels were increased by RIC at 2 h, 24 h, 48 h and 7 d. The expression of the upstream regulator Nrf2 and a further antioxidant gene, NQO1, were also induced.

#### 3.1.5. Intestine

In a direct comparison of ischemic preconditioning versus invasive Pre-RIC, similar protective effects for both treatments on cold I/R injury in a small bowel transplantation model in rats was shown [143]. Ischemic preconditioning of the superior mesenteric artery for 10 min ischemia and 30 min of reperfusion or Pre-RIC with three cycles of 15 min ischemia and 15 min reperfusion of the donors infrarenal aorta before graft extraction reduced damage of intestine 3 h after transplantation. After 6 h of transplantation, damage markers dropped down and achieved similar levels as control. The same pattern was seen for HO-1 expression indicating that HO-1 is involved in conditioning induced protection.

#### 3.1.6. Various Organs

An elegant approach to analyze HO-1 expression in different organs due to RIC was done by Cremers et al. measuring HO-1 promotor activity and HO-1 mRNA in HO-1 luc transgenic mice [144]. These mice carry a transgene consisting of the full-length mouse HO-1 promotor fused to the reporter gene luciferase (luc). Hind limb Pre-RIC with three cycles of 4 min ischemia interspersed with 4 min reperfusion (a protocol for potent protection in a kidney injury model [145]) induced significantly HO-1 promotor activity in the dorsal region and in the kidneys 6h and 24 h after RIC measured with an in vivo Imaging System. HO-1 mRNA levels in ligated muscles, kidney and heart increased 6 h after RIC and dropped to basal levels after 24 h. In contrast, RIC neither increased HO-1 mRNA levels in the skin nor improved cutaneous wound repair after excisional skin injury [144].

Taken together, numerous organs like liver, kidneys, heart and lungs benefit from the protection by Pre-RIC. This protection is associated with an increase in HO-1 expression starting early after the intervention (2 h) and remaining at high levels for at least 24 h. Experiments with HO-1 inhibitors, such as SnPP-IX, inhibiting the protective effects underline the importance of HO-1 increase. Furthermore, Nrf2 translocation seems to be a main mechanism of HO-1 regulation. The protective effect of HO-1 seems to be mediated, at least in part, by induction of autophagy.

### 3.2. Remote Ischemic Postconditioning (Post-RIC)

The first study investigating regulatory effects of Post-RIC on HO-1 was done by Zhang et al. [146]. A quite unusual bilaterally hind limb RIC protocol was used by a combination of per-conditioning with onset of ischemia and daily Post-RIC (7 d) in a retinal ischemic mouse model. The non-invasive RIC procedure (three cycles of 10 min I/R) with tourniquet induces a neuroprotective effect via reduction of retinal tissue damage and a further induction of ischemia-induced increase of Nrf2 and HO-1 protein levels. The authors suggested that the retinal protective effect of RIC is mediated by induction of the antioxidative signaling cascade Nrf2/HO-1, resulting in reduced neuronal damage [146].

A neuroprotective effect by limb Post-RIC via HO-1 upregulation was also described by Ramagiri and Taliyan [147]. After induction of cerebral I/R injury with a bilateral common carotid occlusion model, rats were postconditioned (three cycles of 10 min I/R) with a tourniquet. Post-RIC restored neurological and cognitive functions and reduced the oxidative stress, neuroinflammation, acetylcholinesterase levels and hippocampal structural abnormalities. I/R injury increased HO-1 protein levels, which were intensified by Post-RIC. SnPP-IX application reversed the HO-1 upregulation and the protective effects of Post-RIC [147].

Gao et al. investigated the Nrf2-pathway as a mechanism of Post-RIC (three cycles of 5 min I/R of left femoral artery) in more detail and could show that Post-RIC seems to be mediated by Nrf2- antioxidant signaling activated via the janus kinase (JAK)/STAT/Akt/endothelial nitric oxide synthase (eNOS) pathway [148]. This group used a cardiac I/R mouse model and demonstrated initially a substantial reduction of infarct size, apoptosis, and cell damage after I/R injury by RIC. Additionally, oxidative stress was reduced reflected in, amongst others, decreased levels of MDA. Post-RIC also increased levels HO-1 protein and nuclear translocation of Nrf2. Due to increased phosphorylation of STAT3 by Post-RIC, the STAT3 inhibitor AG6490 was applied before RIC. This inhibitor reversed all protective effects (as well as increased HO-1 levels) of Post-RIC indicating a STAT3 depending pathway. In addition, Post-RIC induced increase of Akt, as well as eNOS phosphorylation was also abolished by AG6450 suggesting a crucial role of downstream targets of STAT3 in the cardioprotective signaling of Post-RIC [148].

As mentioned in the section above, two studies directly compared Pre- and Post-RIC on different models of liver injury. Zheng et al. showed a protective effect for both conditioning time points on acetaminophen-induced acute liver injury in mice [135]. The hepatotoxicity was reduced by inhibiting inflammation and oxidative stress. The latter was represented among others by further elevated HO-1 protein amounts via RIC. ZnPP-IX application counteracted the positive effects of RIC, whereas the HO-1 inducer Cobalt protoporphyrin IX could mimic the reducing effect of Post-RIC on ALT and AST activities as marker of liver damage.

In line with the results of Zheng et al. [135], Czigany et al. demonstrated also for both Pre- and Post-RIC a protective effect in an arterialized orthotopic liver transplantation (OLT) model in rats, but for Post-RIC in a weaker extent [136]. Interestingly, the transplantation-induced increase of HO-1 mRNA expression in the liver tissue was further elevated by Pre-RIC, but not by Post-RIC. For Post-RIC, the infrarenal aorta of recipient rats were clamped with four cycles of 5min ischemia and 5 min reperfusion.

In summary, the described studies show that Post-RIC is also a viable and efficacious tool to induce neuro-, cardio- and hepatoprotective effects. The mechanism seems to be similar to those of Pre-RIC: increased HO-1 expression mediated by Nrf2 is essential for protection. With regard to translation into the clinics, a non-invasive stimulus for Pre- and Post-RIC seems to be suitable to induce HO-1 expression resulting in protection.

## 4. Conclusions

Under numerous pathophysiological conditions, upregulation of HO-1 has been shown to be beneficial. However, under some circumstances, HO-1 upregulation might also prove detrimental. The extent of HO-1 upregulation, the nature of the stress event, the cellular environment and possibly further factors determine the outcome. Anesthesia-related drugs and remote ischemic stimuli represent suitable conditioning strategies to induce heme oxygenase-1, either on its own or in combination with the harmful event as part of an adaptive stress response. The favorable clinical safety profile of both forms of conditioning to induce tolerance to the tissue at risk is appealing. The use of sub-anesthetic doses of anesthetics would open new areas of application. Targeting the Nrf2/HO-1 axis and activation of pro survival MAPK, PI3K/Akt and STAT3 signaling pathways seem to be key elements in mediating conditioning effects. The regulation of HO-1 by microRNAs and the role of autophagy in the context of conditioning warrant deeper investigation.

## Supplementary Materials

The following are available online at https://www.mdpi.com/2076-3921/8/9/403/s1, Table S1: Conditioning with anesthetics and its effect on HO-1 and organ protection, Table S2: Remote ischemic conditioning and its effect on HO-1 and organ protection.
